# Factors associated with death among postpartum women with COVID-19: a Brazilian population-based study*

**DOI:** 10.1590/1518-8345.5446.3507

**Published:** 2021-11-19

**Authors:** Anelise de Toledo Bonatti, Nathassia Miller, Maria Antonieta de Barros Leite Carvalhaes, Rodrigo Jensen, Cristina Maria Garcia de Lima Parada

**Affiliations:** 1 Universidade Estadual Paulista “Júlio de Mesquita Filho”, Faculdade de Medicina de Botucatu, Botucatu, SP, Brazil.

**Keywords:** Coronavirus Infections, Pandemic, Postpartum Period, Maternal Death, Clinical Evolution, Nursing Care, Infecciones por Coronavirus, Pandemia, Muerte Materna, Periodo Posparto, Evolución Clínica, Atención de Enfermería, Infecções por Coronavírus, Pandemia, Morte Materna, Período Pós-Parto, Evolução Clínica, Cuidados de Enfermagem

## Abstract

**Objective:**

to identify the factors associated with death due to COVID-19 among Brazilian postpartum women in the first five months of the pandemic and five subsequent months, and describe the sociodemographic and clinical characteristics of postpartum women who developed the disease.

**Method:**

cross-sectional population-based study using a secondary database available in the *Sistema de Informação de Vigilância Epidemiológica da Gripe -SIVEP-Gripe* (Influenza Epidemiological Surveillance Information System), Brazilian Ministry of Health. A total of 869 postpartum women were included, and the analysis considered the first five months of the pandemic and subsequent five months. Association between the variables of interest and outcome (death due to COVID-19/cure) was investigated using logistic regression.

**Results:**

most participants were aged between 20 and 34, of mixed race or Caucasian, and lived in the urban/peri-urban area. The proportion of deaths was 20.2% in the first period and 11.2% in the second. The likelihood of death increased in both periods due to the presence of respiratory signs and symptoms: dyspnea, respiratory distress, and oxygen saturation below 95%, in addition to the need for ventilatory support and intensive care.

**Conclusion:**

the proportion of deaths among postpartum women was high and decreased in the second period under study. Respiratory signs and symptoms, mechanical ventilation, and intensive care were associated with death in both periods.

## Abstract (es)

**Objetivo:**

identificar los factores asociados a muerte por COVID-19 durante el puerperio en brasileñas, en los primeros cinco meses de la pandemia y en los cinco meses posteriores y, describir las características sociodemográficas y clínicas de puérperas que desarrollaron la enfermedad.

**Método:**

estudio transversal, de base poblacional, con datos secundarios del *Sistema de Informação de Vigilância Epidemiológica da Gripe - SIVEP-Gripe* (Sistema de Información de Vigilancia Epidemiológica de la Gripe), del Ministerio de la Salud de Brasil. Fueron incluidas 869 puérperas y el análisis consideró los primeros cinco meses de la pandemia y los cinco meses posteriores. La asociación entre las variables de interés y el resultado (muerte/cura por COVID-19) fue investigada por regresión logística.

**Resultados:**

la mayoría de las puérperas tenía entre 20 y 34 años, color de piel parda/blanca y residía en zona urbana/periurbana. La proporción de muertes fue de 20,2% en el primer período y 11,2% en el segundo. En los dos períodos - *aumento* más las probabilidades de *morir* - la presencia de señales y síntomas respiratorios (disnea, incomodidad respiratoria y saturación de oxígeno inferior a 95%), así como necesitar de soporte ventilatorio y terapia intensiva.

**Conclusión:**

la proporción de muertes entre puérperas fue elevada, con reducción en el segundo período estudiado. Se asociaron a la muerte señales y síntomas respiratorios, la necesidad de ventilación mecánica y de terapia intensiva, en los dos períodos analizados.

## Abstract (pt)

**Objetivo:**

identificar os fatores associados ao óbito por COVID-19 entre puérperas brasileiras, nos primeiros cinco meses da pandemia e nos cinco meses posteriores e descrever as características sociodemográficas e clínicas de puérperas que desenvolveram a doença.

**Método:**

estudo transversal, de base populacional, com dados secundários do Sistema de Informação de Vigilância Epidemiológica da Gripe, disponibilizados pelo Ministério da Saúde do Brasil. Foram incluídas 869 puérperas e a análise considerou os primeiros cinco meses da pandemia e os cinco meses posteriores. A associação entre as variáveis de interesse e o desfecho (óbito/cura por COVID-19) foi investigada por regressão logística.

**Resultados:**

a maioria das puérperas tinha entre 20 e 34 anos, cor da pele parda/branca e residia em zona urbana/periurbana. A proporção de óbitos foi de 20,2% no primeiro período e 11,2% no segundo. Em ambos os períodos, aumentou a chance de evolução para óbito a presença de sinais e sintomas respiratórios: dispneia, desconforto respiratório e saturação de oxigênio inferior a 95%, assim como necessitar de suporte ventilatório e terapia intensiva.

**Conclusão:**

a proporção de óbitos entre puérperas foi elevada, com redução no segundo período estudado. Associaram-se ao óbito sinais e sintomas respiratórios, necessidade de ventilação mecânica e de terapia intensiva, em ambos os períodos analisados.

## Introduction

COVID-19 has impacted nations differently depending on each country’s economic, social, and political context^(1)^. However, the occurrence of this disease in the pregnancy-puerperal cycle has resulted in adverse outcomes not only in low-income countries, with restricted resources and precarious health systems, but also in developed and well-structured countries with traditionally low maternal mortality rates^(2)^. Hence, the pandemic has evidenced an important crisis in the health field in different contexts while revealing the relevance of health workers in general, though of nurses in particular; the competencies of nurses are essential at all levels of healthcare services^(3)^.

From 2020 onwards, the United States presented the highest rates of Covid-19 worldwide due to three overlapping epidemic waves. Brazil, the United Kingdom, Italy, and Spain stood out as they presented a similar pattern of high incidence and mortality; in Brazil, the disease evolved heterogeneously both between and within states. In 2020, Brazil totaled 7,714,819 cases and 195,742 deaths over the 44 epidemiological weeks. An unequal pattern was also verified in the distribution of more complex health services and quality care^(4)^.

Similar to other population groups, the way the disease progressed among pregnant and postpartum women varied. A systematic review addressing the occurrence of COVID-19 in the prenatal period reports the presence of both asymptomatic cases and clinical conditions of varying levels of severity, including typical airway infection and even nonspecific manifestations, with systemic and/or gastrointestinal symptoms^(5)^. The most common clinical repercussions for the population, in general, include difficulty breathing or increased respiratory rate, oxygen saturation below 95%, and the worsening of underlying diseases and hypotension^(6-7)^; one in six infected people develop the severe form of the disease^(8-9)^. However, thus far, there is not a study specifically describing how the disease progresses among postpartum women.

At the beginning of the pandemic, pregnant and postpartum women did not seem to be any more vulnerable to the severe form of the disease than the population in general^(10-11)^. However, more recently, recommendations were revised based on studies reporting that pregnancy and postpartum may represent additional risks to mothers and infants^(7,12-13)^, probably because of the physiological changes inherent to pregnancy. Immunodeficiency, increased susceptibility to respiratory pathogens, and changes in organic responses to viral infections^(14-17)^ result in a greater risk of invasive ventilation and admittance into an Intensive Care Unit (ICU)^(18-19)^, and even death^(2)^.

A Swedish report showed that the maternal group was five times more likely to be admitted to an ICU than non-pregnant women, though the risk of death was the same for both groups^(20)^. In comparison, a Brazilian study found that postpartum women were two times and half times more likely to experience adverse effects than pregnant women^(21)^. For this reason, pregnant and postpartum women infected with COVID-19 up to two weeks postpartum deserve special attention, as do women who experienced a miscarriage or fetal loss^(7)^.

Brazil, a country that usually presents high maternal mortality rates, has witnessed an increase in the number of deaths during the pregnant-puerperal cycle since the beginning of the COVID-19 pandemic^(15)^, surpassing other countries, as international reports show^(22-25)^. However, research usually addresses pregnant women, and few studies focus on the clinical outcome of the SARS-CoV-2 during postpartum to contribute to therapeutic and preventive decisions directed to this group.

Understanding how COVID-19 progresses specifically among postpartum women is a gap this study is intended to fill in by achieving the following objectives: 1 - identify the factors associated with death due to COVID-19 among Brazilian postpartum women in the first five months of the pandemic and subsequent five months; and 2 - describe the sociodemographic and clinical characteristics of postpartum women who developed the disease.

These two periods were chosen because of the way deaths caused by COVID-19 progressed in Brazil in 2020. An expressive and constant increase in deaths was observed since the first deaths occurred in early March, reaching the worst result on June 4^th^ (1,473 deaths). From this peak onwards, the decrease was progressive, albeit slow, culminating in 128 deaths on November 8^th^, with a subsequent new progressive increase^(26)^. The hypothesis tested here is that the profile of postpartum women, and consequently, the factors associated with death, change over time due to the changes implemented in preventive measures and treatment. Therefore, the deaths among postpartum women are also expected to decrease over time with the emergence of evidence that enables improved treatments.

## Method

### Study design

This is a cross-sectional, population-based study.

### Study setting

Brazil is a continent-spanning country^(27)^ that initiated a demographic transition in the 1970s by decreasing mortality and fertility rates, influencing its age structure that resulted in population aging^(28)^.

Brazil consolidated an extensive primary healthcare network in the last three decades, which is the main gateway to its universal public health system, called Unified Health System (SUS). However, SUS faces some issues such as underfunding, the changes already mentioned in the population’s epidemiological profile, the current economic crisis and the consequent impoverishment of the population, and the emergence of patients with a new profile, that is, more attentive and demanding, among other aspects^(27)^.

As a member of the United Nations, Brazil’s priority is to decrease maternal mortality rates. However, it has not yet achieved what the Millennium Development Goals (MDG) propose: child mortality decreased by 55% between 1990-2011, from 141 to 64 deaths *per* 100,000 live births, however, far from the 35 deaths *per* 100,000 live births proposed for the country to achieve by 2015^(29)^. Hence, maternal mortality remains high^(30)^.

### Selection criteria

Due to the high sensitivity and specificity of COVID-19 testing^(31)^, the study included postpartum women with laboratory confirmation of SARS-CoV-2 by real-Time Polymerase Chain Reaction (RT-PCR) and confirmation regarding the outcome (death yes, no) at two different points in time: from March 22^nd^, 2020 to August 8^th^, 2020 and from August 9^th^, 2020 to January 2^nd^, 2021, totaling 19 and 20 epidemiological weeks, respectively. Those classified as pregnant and postpartum women were excluded, as were those outside the age range considered in the definition of childbearing age (from 10 to 49 years old).

### Data collection

Data were collected from a secondary population-based dataset available in the *Sistema de Informação de Vigilância Epidemiológica da Gripe - SIVEP-Gripe* (Influenza Epidemiological Surveillance Information System), INFLUD-Aug-10-2020 and INFLUD-Jan-25-2021 databases, Brazilian Ministry of Health (available at: https://opendatasus.saude.gov.br/dataset/bd-srag-2020) These include from the 13^th^ (March 22^nd^-28^th^, 2020) to the 32^nd^ epidemiological week (August 2^nd^-8^th^, 2020), and from the 33^rd^ (August 9^th^-15^th^, 2020) to the 53^rd^ epidemiological week (December 27^th^, 2020 to January 2^nd^, 2021), and included data from the entire country. Data collection ceased in January 2021.

The following procedure was used to select the study sample aiming to obtain a qualified database: we initiated with the complete database (575,935 cases on August 8^th^, 2020 and 1,048,576 cases on January 2^nd^, 2021) and then selected from the 13^th^ to 32^nd^ (563,851 cases) and from 33^rd^ to 53^rd^ (473,969 cases) epidemiological weeks. Next, women up to 45 days postpartum (2,561 and 1,606 cases) were selected, followed by those infected with COVID-19 (1,001 and 634 cases). Some of these women were referred to as pregnant and postpartum, but those classified as postpartum only remained (725 and 523 cases). Next, only women aged between 10 and 49 years old (649 and 391 cases) were selected, and finally, the cases whose outcome was reported (death yes, no), which is outcome addressed in this study, remained in the sample (540 and 329 cases).

### Study’s variables

The outcome variable is death due to COVID-19 (death-cure). The independent variables were:

- **sociodemographic variables:** age in years (10-19, 20-34, 35 or older), race (Caucasian, Afro-descendant, Asian, mixed-race, indigenous), region of residence (Southeast, Northeast, North, Mid-West, South), and area of residence (urban or peri-urban, rural);
- **variables related to infection and disease severity (yes, no):** nosocomial case, hospitalization, ICU admittance, and ventilatory support.
- **comorbidities (yes, no):** heart disease, hematologic disease, Down Syndrome, asthma, diabetes, neurological disease, lung disease, immunodeficiency, nephropathy, and obesity. Considering that *SIVEP-Gripe* requires that such an event be reported, this variable was considered dichotomous; hence the cases in which this information was not provided (blank) were included in the No group;
- **respiratory signs and symptoms (yes, no):** dyspnea, respiratory distress, and oxygen saturation below 95%. Number of respiratory signs and symptoms presented (0, 1, 2, 3);
- **other clinical signs and symptoms (yes, no):** cough, fever, odynophagia, headache, runny nose, anosmia, diarrhea, nausea and vomiting, myalgia, ageusia, weakness, chills, nasal congestion, abdominal pain, back and lower back pain, chest pain, fatigue, skin rash, tachycardia, inappetence, malaise, shock, cyanosis of the extremities, pallor, arthralgia, hemoptysis, dizziness, agitation, and the number of clinical signs and symptoms (0-1, 2-3, 4 or more). Considering that *SIVEP-Gripe* also requires that such an event be reported, this variable was considered dichotomous; hence the cases in which this information was not provided (blank) were included in the No group.

### Data analysis

Initially, a descriptive analysis of the variables concerning sociodemographic data, infection, comorbidities, and respiratory and clinical signs and symptoms at the time the disease and its progression were reported. Next, the association between the variables of interest and the outcome was investigated using logistic regression, estimating odds ratios and respective 95% confidence intervals; the significance level was set at p<0.05. Only comorbidities with a proportion higher than 1.0% were included in the analysis, and clinical signs and symptoms were analyzed considering the total number presented in both epidemiological weeks: 13^th^-32^nd^ and 33^rd^-53^rd^. No data imputation methods were used, even for variables with more than 20% of missing data, such as race and nosocomial cases. The analysis was performed using SPSS v.21.0.

### Ethical aspects

Ethical guidelines were complied with according to Resolution No. 510, from April 7^th^, 2016, single paragraph, Brazilian National Health Council, which states that studies using publicly available information do not need to be registered nor assessed by an Institutional Review Board, according to Law No. 12,527, November 18^th^, 2011^(32)^.

Note that the database used is publicly available and does not report the names of the participants, and there is no possibility to individually identify any of the participants so that anonymity is ensured. Thus, since this study used a publicly available database, there was no need to be submitted to an Institutional Review Board.

## Results

Sociodemographic characteristics and those concerning the infection of postpartum women are presented in Table 1. Most of the women addressed in both periods were aged between 20 and 34, were Mixed-race and Caucasian, lived in the urban or peri-urban area, acquired non-nosocomial infection, were not admitted to an ICU, and did not require mechanical support. There was considerable increase in the number of cases in the Mid-West and South, almost all were hospitalized and 20.2% and 11.2% of the cases progressed to death in the first and second periods, respectively, i.e., deaths decreased by 44.6%.

**Table 1:** Sociodemographic characteristics and those concerning COVID-19, and the progression of postpartum women in the 13^th^-32^nd^ weeks (n=540) and 33^rd^-53^rd^ weeks (n=329). Brazil, 2020-2021

Characteristics	13^th^-32^nd^ Weeks	33^rd^-53^rd^ Weeks
	n (%)	n (%)
**Age (years)** Up to 19 20-34 35 or older	56 (10.4) 337 (62.4) 147 (27.2)	33 (10.0) 193 (58.7) 103 (31.3)
**Race** Caucasian Mixed Afro-descendent Asian Indigenous Missing	115 (21.3) 283 (52.4) 34 (6.3) 4 (0.7) 3 (0.6) 101 (18.7)	120 (36.5) 133 (40.4) 18 (5.5) 1 (0.3) 6 (1.8) 51 (15.5)
**Region of residence** Southeast Northeast North Mid-West South	216 (40.0) 188 (34.8) 72 (13.4) 38 (7.0) 26 (4.8)	120 (36.5) 59 (17.9) 40 (12.2) 51 (15.5) 59 (17.9)
**Area of residence** Urban or peri-urban Rural Missing	462 (85.6) 31 (5.7) 47 (8.7)	280 (85.1) 15 (4.6) 34 (10.3)
**Nosocomial case** Yes No Missing	21 (3.9) 395 (73.1) 124 (23.0)	13 (4.0) 240 (72.9) 76 (23.1)
**Hospitalization** Yes No Missing	523 (96.9) 13 (2.4) 4 (0.7)	324 (98.5) 2 (0.6) 3 (0.9)
**Admitted to an ICU** Yes No Missing	175 (32.4) 326 (60.4) 39 (7.2)	107 (32.5) 204 (62.0) 18 (5.5)
**Ventilatory support** Yes No Missing	212 (39.3) 284 (52.6) 44 (8.1)	150 (45.6) 153 (46.5) 26 (7.9)
**Outcome** Cure Death	431 (79.8) 109 (20.2)	292 (88.8) 37 (11.2)

Table 2 presents the participants’ comorbidities. Both groups presented a proportion above 1.0% of the following comorbidities: heart disease, diabetes, obesity, asthma, immunodeficiency, and nephropathy. Hematologic diseases also reached a proportion above 1.0%, but only in the group within the 33^rd^-53^rd^ weeks.

**Table 2:** Absolute and relative frequencies of comorbidities among postpartum women with COVID-19 considering the 13^th^-32^nd^ weeks (n=540) and 33^rd^-53^rd^ weeks (n=329). Brazil, 2020-2021

Comorbidities	13^th^-32^nd^ Weeks	33^rd^-53^rd^ Weeks
	n (%)	n (%)
**Heart disease** Yes No	47 (8.7) 493 (91.3)	31 (9.4) 298 (91.6)
**Diabetes** Yes No	33 (6.1) 507 (93.9)	30 (9.1) 299 (90.0)
**Obesity** Yes No	18 (3.3) 522 (96.7)	21 (6.4) 308 (93.6)
**Asthma** Yes No	13 (2.4) 527 (97.6)	17 (5.2) 312 (94.8)
**Immunodeficiency** Yes No	9 (1.7) 531 (98.3)	5 (1.5) 324 (98.5)
**Nephropathy** Yes No	7 (1.3) 533 (98.7)	4 (1.2) 325 (98.8)
**Down Syndrome** Yes No	3 (0.6) 537 (99.4)	2 (0.6) 327 (99.4)
**Hematologic diseases** Yes No	2 (0.4) 538 (99.6)	4 (1.2) 325 (98.8)
**Neurological disorders** Yes No	3 (0.6) 537 (99.4)	2 (0.6) 327 (99.4)
**Lung disease** Yes No	2 (0.4) 538 (99.6)	3 (0.9) 326 (99.1)

Table 3 presents signs and symptoms: 60.7% of the participants in the first period and 58.6% in the second period presented one or more respiratory symptoms. Dyspnea was the most frequent respiratory symptom, affecting 47.4% and 44.4% of the women in the first and second periods, respectively; 10% in the first period and 48.9% in the second period presented four or more clinical symptoms, the most frequent were cough, fever, odynophagia, headache, running nose, anosmia, diarrhea, and myalgia; present in at least 5% of the cases in both periods.

**Table 3:** Frequency of postpartum women, according to signs and symptoms presented at the time of notification due to COVID-19 in the 13^th^-32^nd^ weeks (n=540) and 33^rd^-53^rd^ weeks (n=329). Brazil, 2020-2021

Variables	13^th^-32^nd^ Weeks	33^rd^-53^rd^ Weeks
	n (%)	n (%)
Number of respiratory SS and SS*		
0	212 (39.3)	136 (41.4)
1	113 (20.9)	58 (17.6)
2	74 (13.7)	60 (18.2)
3	141 (26.1)	75 (22.8)
**Respiratory SS and SS** *		
Dyspnea	256 (47.4)	146 (44.4)
Respiratory discomfort	232 (43.0)	143 (43.5)
Oxygen saturation <95%	196 (36.3)	114 (34.6)
**Number of other clinical SS and SS** *		
0-1	248 (45.9)	51 (15.5)
2-3	238 (44.1)	117 (35.6)
4 or more	54 (10.0)	161 (48.9)
**Other clinical SS and SS** *		
Cough	314 (58.1)	180 (54.7)
Fever	313 (58.0)	148 (45.0)
Odynophagia	114 (21.1)	55 (16.7)
Headache	53 (9.8)	40 (12.1)
Running nose	48 (8.9)	18 (5.5)
Anosmia	47 (8.7)	55 (16.7)
Diarrhea	47 (8.7)	23 (7.0)
Nausea and vomit	40 (7.4)	15 (4.5)
Myalgia	37 (6.8)	18 (5.5)
Ageusia	18 (3.3)	46 (14.0)
Weakness	11 (2.0)	7 (2.1)
Shiver	8 (1.5)	6 (1.8)
Nasal congestion	7 (1.3)	6 (1.8)
Back and lower back pain	5 (0.9)	0 (0.0)
Chest pain	5 (0.9)	8 (2.4)
Tiredness	4 (0.7)	1 (0.3)
Skin rash	3 (0.6)	0 (0.0)
Tachycardia	3 (0.6)	3 (0.9)
Inappetence	2 (0.4)	2 (0.6)
Malaise	2 (0.4)	4 (1.2)
Shock	1 (0.2)	0 (0.0)
Cyanosis extremities	1 (0.2)	0 (0.0)
Pallor	1 (0.2)	0 (0.0)
Arthralgia	1 (0.2)	3 (0.9)
Hemoptysis	1 (0.2)	2 (0.6)
Dizziness	1 (0.2)	1 (0.3)
Agitation	1 (0.2)	0 (0.0)

* Signs and symptoms

Table 4 presents the results of the logistic regression analyses concerning sociodemographic data, type of infection, and presence of comorbidities. In the first period, only postpartum women aged 35 years old or older infected with COVID-19, were twice more likely to die (OR=1.90; CI95%=1.21-2.99). In the second period, Afro-descendant women with COVID-19 were four times more likely to die than their Caucasian counterparts (OR=4.23; CI95%=1.25-14.29) while living in the Northeast increased eight times the likelihood of death (OR=8.05; CI95%=1.72-37.51) compared to those living in the South.

**Table 4:** Results of the logistic regression analysis to estimate the likelihood of death among postpartum women due to COVID-19, concerning sociodemographic data, infection, and comorbidities in the 13^th^-32^nd^ weeks (n=540)and 33^rd^-53^rd^ weeks (n=329). Brazil, 2020-2021

Variables	13^th^-32^nd^ Weeks				33^rd^-53^rd^ Weeks			95%CI^†^
	Death n(%)	No death n(%)	OR*	95%CI^†^	Death n(%)	No death n(%)	OR*	
**Sociodemographic**								
Age (years) 20-34 10-19 35 or older	60(17.8) 6(10.7) 43(29.3)	277(82.2) 50(89.3) 104(70.7)	- 0.55 1.90	- 0.22-1.35 1.21-2.99	1(3.0) 25(13.0) 11(10.7)	32(97.0) 168(87.0) 92(89.3)	- 4.76 1.24	- 0.62-36.41 0.58-2.64
Race Caucasian Mixed Afro-descendant Asian Indigenous	25(21.7) 60(21.2) 13(38.2) 1(25.0) 1(33.3)	90(78.3) 223(78.8) 21(61.8) 3(75.0) 2(66.7)	- 0.96 2.29 1.20 1.80	- 0.57-1.64 0.98-5.06 0.12-12.0 0.15-20.6	10(8.3) 18(13.5) 5(27.8) - -	110(91.7) 115(86.5) 13(72.2) 1(100.0) 6(100.0)	- 1.72 4.23 - -	- 0.76-3.89 1.25-14.29 - -
Region South Southeast Mid-West Northeast North	7(26.9) 43(19.9) 7(18.4) 34(18.1) 18(25.0)	19(73.1) 173(80.1) 31(81.6) 154(81.9) 54(75.0)	- 0.67 0.61 0.59 0.90	- 0.26-1.70 0.18-2.02 0.23-1.53 0.32-2.50	2(3.4) 15(12.5) 3(5.9) 13(22.0) 4(10.0)	57(96.6) 105(87.5) 48(94.1) 46(78.0) 36(90.0)	- 4.07 1.78 8.05 3.16	- 0.89-18.43 0.28-11.10 1.72-37.51 0.55-18.18
Urban area Yes No	93(20.1) 7(22.6)	369(79.9) 24(77.4)	0.86	0.36-2.06	29(10.4) 3(20.0)	251(89.6) 12(80.0)	0.46	0.12-1.73
**Nosocomial Infection**								
Yes No	5(23.8) 54(17.6)	16(76.2) 316(80.0)	1.25	0.44-3.51	1(7.7) 28(11.7)	12(92.3) 212(88.3)	0.63	0.07-5.03
**Comorbidities**								
Heart disease Yes No	11(23.4) 98(19.9)	36(76.6) 395(80.1)	1.23	0.60-2.50	4(12.9) 33(11.1)	27(87.1) 265(88.9)	1.19	0.39-3.61
Diabetes Yes No	10(30.3) 99(19.5)	23(69.7) 408(80.5)	1.79	0.82-3.88	4(13.3) 33(11.0)	26(86.7) 266(89.0)	1.24	0.40-3.77
Obesity Yes No	4(22.2) 105(20.1)	14(77.8) 417(79.9)	1.13	0.36-3.51	4(19.0) 33(10.7)	17(81.0) 275(89.3)	1.96	0.62-6.17
Asthma Yes No	4(30.8) 105(19.9)	9(69.2) 422(80.1)	1.78	0.54-5.91	1(5.9) 36(11.5)	16(94.1) 276(88.5)	0.47	0.06-3.72
Immunodeficiency								
Yes No	3(33.3) 106(20.0)	6(66.7) 425(80.0)	2.00	0.49-8.14	0(0.0) 37(11.4)	5(100.0) 287(88.6)	-	-
Nephropathy Yes No	3(42.9) 106(19.9)	4(57.1) 427(80.1)	3.02	0.66-13.70	0(0.0) 37(11.4)	4(100.0) 288(88.6)	-	-

* *Odds Ratio*;

† 95% Confidence Interval

Table 5 presents the results of the analyses concerning symptomatology and severity. Postpartum women diagnosed with COVID-19 presenting respiratory signs and symptoms were more likely to die both in the first and second periods, respectively: dyspnea (OR=5.36; 95%CI=3.06-9.39 and OR=8.34; 95%CI=2.85-24.38), respiratory distress (OR=5.01; 95%CI=2.96-8.47 and OR=7.38; 95%CI=2.50-21.79) and oxygen saturation below 95% (OR=8.51; 95%CI=4.87-14.88 and OR=4.14; 95%CI=1.76-9.75). As the number of respiratory symptoms increased, the magnitude of association increased as well.

**Table 5:** Results of the logistic regression analysis to estimate the likelihood of death among postpartum women due to COVID-19, concerning symptomatology and severity, 13^th^-32^nd^ weeks (n=540) and 33^rd^-53^rd^ weeks (n=329). Brazil, 2020-2021

Variables	13^th^-32^nd^ Weeks				33^rd^-53^rd^ Weeks			95%CI^†^
	Death n(%)	No death n(%)	OR*	95%CI^†^	Death n(%)	No death n(%)	OR*	
**Respiratory signs and symptoms**								
Dyspnea Yes No	83(32.4) 26(9.1)	173(67.6) 258(90.9)	5.36	3.06-9.39	30(20.5) 7(3.8)	116(79.5) 176(96.2)	6.50	2.76-15.21
Respiratory distress Yes No	80(34.5) 29(9.4)	152(65.5) 279(90.6)	5.01	2.96-8.47	26(18.2) 11(5.9)	117(81.8) 175(94.1)	3.53	1.68-7.43
O2 saturation <95% Yes No	79(72.5) 30(27.5)	117(27.1) 314(72.9)	8.51	4.87-14.88	21(18.4) 16(7.4)	93(81.6) 199(92.6)	2.80	1.40-5.62
Total Number 0 1 2 3	8(3.8) 24(21.2) 13(17.6) 64(45.4)	204(96.2) 89(78.8) 61(82.4) 77(54.6)	- 6.87 5.43 21.19	- 2.97-15.89 2.15-13.71 9.71-46.25	4(2.9) 5(8.6) 12(20.0) 16(21.3)	132(97.1) 53(91.4) 48(80.0) 59(78.7)	- 3.11 8.25 8.94	- 0.80-12.04 2.53-26.81 2.86-27.92
**Number of clinical signs and symptoms**								
0-1 2-3 4 or more	56(22.6) 48(20.2) 5(9.3)	192(77.4) 190(79.8) 49(90.7)	- 0.86 0.35	- 0.56-1.33 0.13-0.92	9(17.6) 14(12.0) 14(8.7)	42(82.4) 103(88.0) 147(91.3)	- 0.63 0.44	- 0.25-1.57 0.18-1.09
**Severity signs**								
Ventilation Yes No	89(42.0) 11(3.9)	123(58.0) 273(96.1)	17.95	9.26-34.80	29(19.3) 5(3.3)	121(80.7) 148(96.7)	7.09	2.66-18.80
ICU^‡^ Yes No	80(45.7) 20(6.1)	95(54.3) 306(93.9)	12.88	7.49-22.14	27(25.2) 8(3.9)	80(74.8) 196(96.1)	8.26	3.60-18.90

* *Odds Ratio*;

† 95% Confidence Interval;

‡ Intensive Care Unit

Association was found between four or more clinical symptoms and death in the first period. Women infected with COVID-19 in this situation were less likely to die than those with fewer symptoms (OR=0.35; 95%CI=0.13-0.92). Regarding the progression of cases, the severity of symptoms was associated with death in both periods, respectively: ventilatory support (OR=17.95; 95%CI=9.26-34.80 and OR=7.09; 95%CI=2.66-18.88) and ICU admittance (OR=12.88; 95%CI=7.49-22.14 and OR=8.26; 95%CI=3.60-18.97) (Table 5).

## Discussion

This study revealed that deaths among postpartum women decreased by 44.6% from the first to the second period. The results regarding the factors associated with this outcome reinforce the importance of respiratory symptomatology in both periods: dyspnea, respiratory distress, and oxygen saturation below 95%, and the need for mechanical ventilation and ICU admittance. On the other hand, women were less likely to die in the first period as the number of clinical symptoms increased. Regarding sociodemographic aspects in the first period, an association was found between being 35 years old or older and death, while in the second period, Afro-descendant women were more likely to die than Caucasians as well as those living in the Northeast, compared to the women living in the South.

The COVID-19 pandemic reached Brazil while the country was still struggling with an exponentially high maternal mortality rate^(14)^. Additionally, the government did not implement a universal testing policy for the obstetric population; only women with symptoms were tested. Hence, despite the high number of cases identified, the number of COVID-19 infections among postpartum women may be underestimated. A Brazilian study conducted between February and June 2020 reported 978 pregnant and postpartum women diagnostic with COVID-19 and 124 maternal deaths, 3.4 times greater than the total of deaths due to COVID-19 reported in the same period worldwide^(14)^. Another Brazilian study found a risk 2.4 times greater of postpartum women experiencing adverse effects than pregnant women^(21)^.

The highest proportion of deaths among postpartum women was reported in the first period, i.e., in the first months of the pandemic when there was little knowledge about managing the disease. However, despite the important decrease in the number of deaths in the subsequent period, mortality was still high. Hence, considering the entire period under study, the proportion of deaths among postpartum women only was higher than that reported by another Brazilian study, which found a mortality rate of 12.7% between February and June 2020^(14)^. This difference may be due to the fact that the database is continually updated, and the outcomes may be reported at any time, whenever there is a closure for cases.

The high proportion of deaths in the postpartum period may be associated with some factors, among which structural deficiencies in Brazilian maternity hospitals; lack of physical, human, and material resources; lack of resources to manage critical and emergency care; and a lack of beds in ICUs, among other barriers impeding access to health care^(21,33)^. Additional aggravating factors include a decrease in the number of prenatal consultations and routine exams after the pandemic, increased social vulnerability, and the fact that the population relaxed social isolation measures prematurely^(21)^.

The likelihood of death among postpartum women increased in both periods when individuals presented dyspnea, respiratory distress, or oxygen saturation below 95%. Uterine growth during pregnancy by itself is an obstacle to the normal process of maternal ventilation, which increases oxygen demand, and thus, as the uterus expands, the diaphragm is displaced so that dyspnea and respiratory distress are expected^(11)^. In the puerperium, physiological adaptations are complex and characterized by involutive phenomena that occur gradually. Therefore, it is necessary to pay attention to respiratory symptomatology in the postpartum and, whenever symptoms are outside normal parameters, they should be rapidly acknowledged for the condition to be timely reversed.

A meta-analysis addressing seven studies established that the most prevalent symptom associated with ICU admittance among COVID-19 patients, and consequently, with the severity of the condition, was dyspnea. Dyspnea increased 6.6 times the likelihood of individuals being admitted to an ICU compared to those without symptoms. The conclusion was that respiratory distress and dyspnea predicted a severe disease progression^(34)^. Unfortunately, there are no data regarding risk factors for death, especially among postpartum women. However, in this study, dyspnea and respiratory distress had an effect in both periods, indicating the relevance of these symptoms for death.

Other studies presented similar results regarding the finding of severe progression of COVID-19, with the association between the need for mechanical ventilation and ICU admittance to death. For example, a Swiss study addressing women aged between 20 and 45 reports increased risk related to ICU admittance and the use of invasive ventilation among pregnant women and up to one week postpartum, compared to non-pregnant women^(2)^. The same was evidenced by an American analysis with 400,000 women who tested positive for COVID-19 and a retrospective multi-center case-control study, in which pregnant women were more likely to be admitted to an ICU, require mechanical ventilation, intubation, and die^(18,35)^.

In the first epidemiological period addressed in this study, the need for ICU admittance and ventilatory support increased 12.8 and 17.9 times the likelihood of death among postpartum women, respectively. In the second period, the likelihood of death was approximately eight and seven times, respectively. Even though there was a decrease between the periods, the figures were still high, mainly because only postpartum women were considered. In a case series that included pregnant women and women in the immediate postpartum, maternal death occurred in 15% of the patients admitted in an ICU due to COVID-19 (PCR confirmed cases) and 25% of those who required invasive mechanical ventilation. The conclusion was that pregnant and postpartum women with COVID-19 admitted to an ICU are at increased risk to die, even when they present no comorbidities^(36)^. In the initial months of the pandemic, 53 women (i.e., pregnant, non-pregnant, and postpartum women) admitted to a Swedish public health institution required intensive care. Even though all the women were discharged from the ICU, the results show that the risk of ICU admittance increased 5.4 (95%CI 2.89-10.08) and 4.0 times (95%CI 1.75-9.14) among pregnant women and women in the early postpartum with COVID-19, respectively, in comparison to non-pregnant women^(20)^. Note that one of this study’s findings cannot be explained: in the first period, the number of clinical signs and symptoms was a protective factor against death among postpartum women diagnosed with COVID-19.

A late pregnancy, traditionally defined as a pregnancy that occurs at the age of 35 or later, is usually considered a risk pregnancy due to the possibility of comorbidities such as hypertension and diabetes^(37)^, diseases associated with COVID-19^(38)^. Hence, future studies can be designed to confirm whether the association between death due to COVID-19 and late postpartum, found in the first period, is an independent event.

Afro-descendant postpartum women living in the Northeast were more likely to die. A potential explanation is that social and racial inequality found among Brazilian regions is mainly related to difficult access to health services, which may predict worse outcomes for COVID-19 patients^(39-40)^. At the base of these disparities are historically structural and social factors that the pandemic made even more apparent and the weakness of the health system, especially regarding the women’s health care structure^(41)^.

Regarding comorbidities, comparing the second to the first period, there were at least twice as many cases of obesity and asthma. This change in profile may result from improved reporting, with a consequent decrease in missing data. Perhaps scientific evidence indicating the importance of these comorbidities in influencing the severe form of COVID-19^(42-43)^ encouraged improved reporting.

This study’s limitations include using a secondary database that contained information that depended on health workers’ reports, i.e., there were missing data impossible to recover. Additionally, because the database is continually updated and data were collected near the last epidemiological week included in the study, some deaths probably were not reported in time, resulting in some level of underestimation. Note, however, that pending cases were excluded to improve data consistency. On the other hand, it is worth noting that a population-based dataset from a continent-spanning country was used, and data accumulated for a period that may be considered too long, that is, from March 2020 to January 2021.

Considering this study’s results and the fact that nurses provide direct assistance in maternity hospitals and Primary Health Care units, it is essential that nurses monitor postpartum women for respiratory symptomatology via telehealth or face-to-face consultations, promoting rapid testing when necessary, and avoiding delays in implementing the appropriate protocol whenever COVID-19 is confirmed. This procedure will interrupt the transmission chain, minimize the worsening of cases, and consequently decrease the need for ventilatory support and ICU admittance, thus, contributing to decreasing maternal mortality.

## Conclusion

Despite the decrease observed in the second epidemiological period, the proportion of deaths among postpartum women was high. In general, considering both the periods, the factors associated with death were related to respiratory signs and symptoms: dyspnea, respiratory distress, and oxygen saturation below 95%. In addition, an association was found between mechanical ventilation and ICU and the progression of cases. As for sociodemographic aspects, being older, Afro-descendant, and living in the Northeast was associated with deaths.
